# Modest alcohol intake and mortality in individuals with elevated alanine aminotransferase levels: a nationwide cohort study

**DOI:** 10.1186/s12916-021-02215-x

**Published:** 2022-01-24

**Authors:** Dong Hyun Sinn, Danbee Kang, Eliseo Guallar, Yun Soo Hong, Juhee Cho, Geum-Youn Gwak

**Affiliations:** 1 Department of Medicine, Samsung Medical Center, Sungkyunkwan University School of Medicine, Seoul, South Korea; 2 Department of Clinical Research Design and Evaluation, SAIHST, Sungkyunkwan University, Seoul, South Korea; 3 Center for Clinical Epidemiology, Samsung Medical Center, Sungkyunkwan University, Seoul, South Korea; 4 Departments of Epidemiology and Medicine, and Welch Center for Prevention, Epidemiology and Clinical Research, Johns Hopkins Medical Institutions, Baltimore, MD USA

**Keywords:** Alcohol, Alanine aminotransferase, Mortality

## Abstract

**Background:**

Alanine aminotransferase (ALT) levels are widely used to screen liver disease, and many asymptomatic individuals show elevated ALT levels. As elevated ALT level indicates liver injury, even a small amount of alcohol intake may be harmful in subjects with elevated ALT levels, but there is limited evidence of the effect of light to moderate amount of alcohol intake in this subgroup.

**Methods:**

A cohort of 367,612 men and women without established liver diseases (including chronic viral hepatitis, alcohol-associated liver disease, cirrhosis, liver transplantation, or rare forms of liver disease) who underwent at least 1 health screening exam between 2009 and 2015 were assessed for liver-related and all-cause mortality. Elevated ALT levels were defined as ≥ 34 U/L for men and 25 U/L for women.

**Results:**

In participants with normal ALT levels, the fully-adjusted hazard ratios (95% CI) for liver-related mortality comparing light and moderate drinkers to non-drinkers were 0.73 (0.51–1.05), and 1.06 (0.73–1.52), respectively. In participants with elevated ALT levels, the corresponding hazard ratios were 1.57 (1.08–2.28), and 2.09 (CI 1.46–2.99), respectively (*p* value for alcohol intake by ALT interaction < 0.01). For all-cause mortality, the fully-adjusted hazard ratios comparing light and moderate drinkers to non-drinkers in participants with normal ALT levels were 0.72 (0.66–0.77), and 0.89 (0.82–0.97), respectively. In participants with elevated ALT levels, the corresponding hazard ratios were 0.93 (0.81–1.08), and 1.31 (1.14–1.50), respectively (*p* value for alcohol intake by ALT interaction < 0.01).

**Conclusions:**

Small amounts of alcohol intake were associated with increased liver-related and all-cause mortality among individuals with elevated ALT levels. Subjects with elevated ALT levels should be advised complete abstinence from alcohol.

**Supplementary Information:**

The online version contains supplementary material available at 10.1186/s12916-021-02215-x.

## Background

Alcohol intake is a major contributor to the global burden of disease [1, 2]. Alcohol intake is an established risk factor for cancer, liver disease, cardiovascular disease, violence, and injuries [2, 3]. However, while the adverse health effects of excessive alcohol intake are widely accepted, there is substantial controversy on the effects of light to moderate alcohol intake. Some studies found lower risk of cardiovascular disease, diabetes, and mortality with light to moderate alcohol intake [4–6], while others found no safe level of alcohol intake [7, 8].

The effects of alcohol may differ according to underlying diseases or conditions. The liver is the main organ that metabolizes alcohol [9], and the effects of light to moderate alcohol intake may differ in individuals with and without liver disease. For example, complete abstinence is recommended for patients with alcohol-associated liver disease, as any amount of alcohol intake can be harmful [10]. Light to moderate alcohol intake was also associated with increased all-cause mortality in patients with chronic viral hepatitis [11], and abstinence is recommended for them as well [12, 13]. However, the health effects of light to moderate alcohol intake in people with nonalcoholic fatty liver disease (NAFLD) or with less prevalent forms of liver disease (e.g., Wilson’s disease, autoimmune, or cryptogenic liver disease) are controversial and information is scarce [14].

Liver injury or liver disease is usually asymptomatic until complications of liver failure or portal hypertension develop [15]. Thus, liver function tests, including bilirubin, albumin, alkaline phosphatase, gamma-glutamyltransferase (GGT), and aminotransferase levels are the mainstay for identifying early liver injury or liver disease [15, 16]. While alkaline phosphatase, GGT, and aspartate aminotransferase (AST) can be produced by organs other than the liver, alanine aminotransferase (ALT) is exclusively produced in hepatocytes and widely used as a specific biomarker of liver injury or liver disease [15, 16]. As serum ALT measurement is relatively inexpensive, it is frequently used to screen for or exclude liver disease [16, 17], and it is common to find asymptomatic individuals with abnormally elevated ALT levels [18]. Subjects with elevated ALT levels are recommended to undergo additional workup to establish the etiology of liver injury [16]. In these subjects, the most common causes of elevated ALT levels are alcohol-associated liver disease, chronic viral hepatitis, and NAFLD [18]. When alcohol-associated liver disease or chronic viral hepatitis are identified as the causes of elevated ALT, current evidence and practice guidelines recommend complete abstinence [10–13]. In subjects with other causes of elevated ALT levels (mostly subjects with NAFLD), it is controversial if they require complete abstinence and information regarding this issue is limited. Thus, we aimed to evaluate the association of light to moderate alcohol intake on liver-related mortality and all-cause mortality among individuals with elevated ALT levels, but excluding those with excessive alcohol intake or viral hepatitis, using a nationwide population-based cohort.

## Methods

### Study population and design

This is a retrospective cohort study based on data from the National Health Insurance Service (NHIS) National Sample Cohort (NHIS-NSC) 2.0, an administrative nationwide cohort which includes a representative 2.2% sample of the Korean population in 2006 [19]. Data can only be accessed by visiting the NHIS datacenter, after approval from data access committee of NHIS (https://nhiss.nhis.or.kr/bd/ab/bdaba001cv.do). This research was conducted using the application number NHIS-2019-2-034. South Korea has a single-payer universal health system. The NHIS maintains claims data on all reimbursed inpatient and outpatient visits, procedures, and prescriptions. In addition, the NHIS-NSC 2.0 includes data from annual or biennial health screening exams provided free of charge by the Ministry of Health and Welfare. Approximately 75% of all eligible persons underwent a health screening exam [20].

**Fig. 1:**
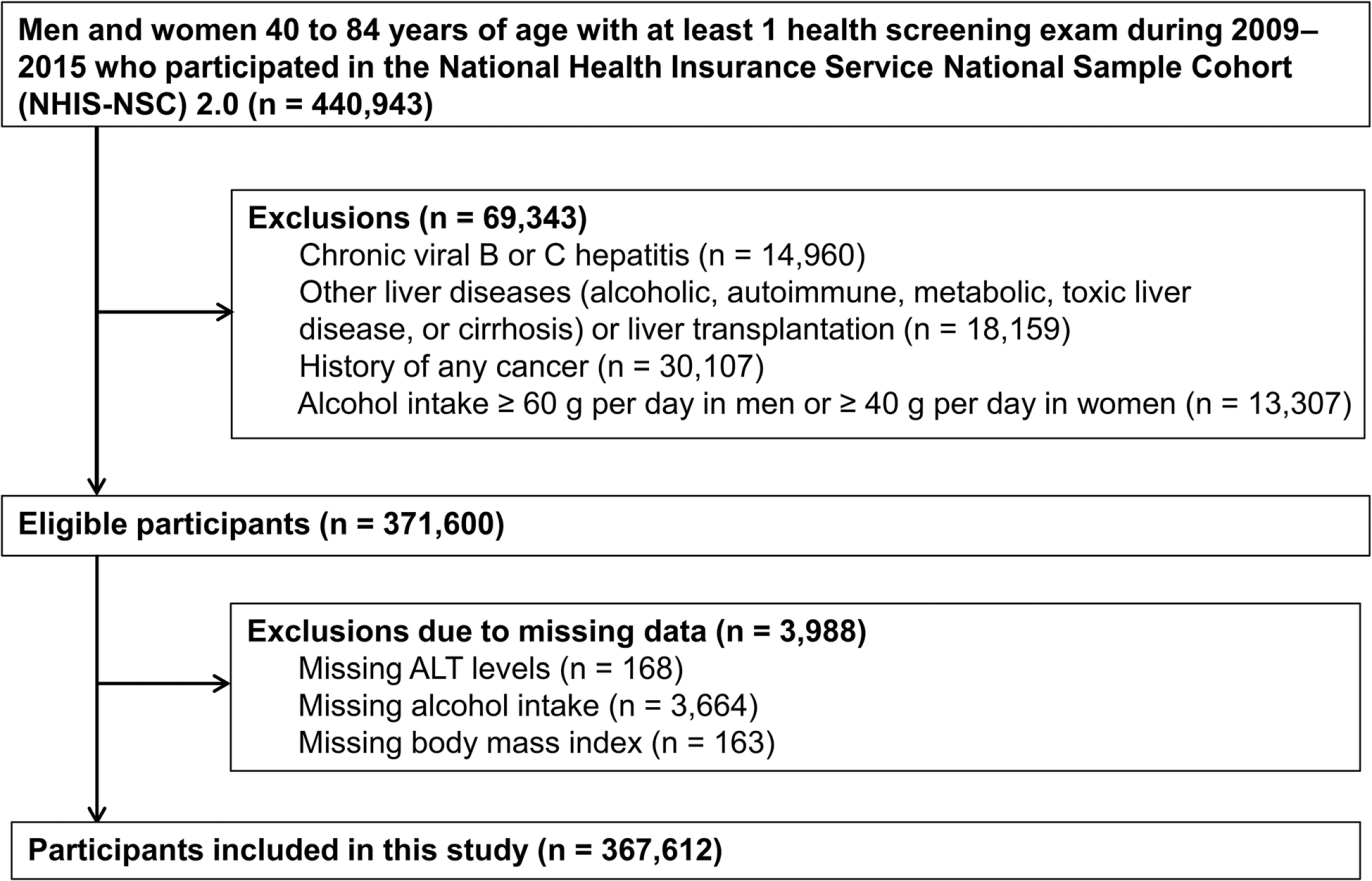
Flowchart of study participants

In this analysis, we included NHIS–NSC 2.0 participants with 40–84 years of age who underwent at least one health screening exam between January 1, 2009, and December 31. 2015 (*N* = 440,943). We selected 2009 as the first year of follow-up because health screening exams were substantially modified in 2009. We then excluded participants with chronic viral hepatitis (defined as any inpatient or outpatient visit with International Classification of Diseases, Tenth Revision [ICD-10] codes B18 or Z225; *N* = 14,960), alcohol-associated liver disease (codes K700-K704, or K709; *N* = 3,768), autoimmune liver disease (codes K754 or K743; *N* = 69), metabolic liver disease (codes E830 or E831; *N* = 12), toxic liver disease (codes K711, K713-K715, or K717; *N* = 44), liver cirrhosis (code K74; *N* = 1,541), liver transplantation (code Z944; *N* = 84), or history of any cancer (any ICD-10 C code; *N* = 30,107). In addition, we excluded participants who, in the health screening exam, reported heavy alcohol intake (defined as ≥ 40 g/day in women and ≥ 60 g/day in men; *N* = 13,307). We further excluded 3,988 participants with missing data on ALT (*N* = 168), alcohol status (*N* = 3664) or body mass index (BMI) (*N* = 163) in the health screening exam, resulting in a final sample of 367,612 individuals (172,287 men and 195,325 women; Fig. 1). The Institutional Review Board of the Samsung Medical Center approved the study and waived the requirement for informed consent because NHIS-NSC 2.0 data were publicly available de-identified data.

### Study variables

The NHIS-NSC2.0 data includes modules on insurance eligibility, medical treatments, and health screening exams. The insurance eligibility module contains information on age, sex, residential area, and income level. The medical treatment module contains information on claims for all inpatient and outpatient diagnoses, procedures, and prescriptions [19], coded using ICD-10 codes and the Korean Drug and Anatomical Therapeutic Chemical Codes [21]. The NHIS routinely audits all claims and the data are considered reliable and have been used in many peer-reviewed publications [22].

The health screening exam module included data from questionnaires, anthropometric measurements, and blood tests to screen for anemia, diabetes, cardiovascular risk factors, kidney disease, and liver disease. If a participant had more than one health screening exam during the study period, we used only data from the initial (baseline) screening exam. The frequency and amount of alcohol drinking and smoking habits were collected by self-administered questionnaires. Current alcohol intake (g/day) was calculated using frequency of drinking (times/week) and amount of alcohol at each occasion. We categorized alcohol intake according to the American Association for the Study of Liver Diseases (AASLD) guidelines [10] into none, light (< 10 g/day in women and < 20 g/day in men), moderate (10–< 40 g/day in women and 20–< 60 g/day in men), and heavy (≥40 g/day in women and ≥60 g/day in men). Smoking status was categorized into never, and ever (past or current) smoker. Height, weight, waist circumference, and blood pressure were measured at the health screening exam. BMI was calculated as weight in kilograms divided by height in meters squared [23]. We classified study participants into two groups based on serum ALT level at the health screening exam: normal (ALT < 34 U/L for men and < 25 U/L for women), and elevated (ALT ≥34 U/L for men and ≥25 U/L for women) [18].

Co-morbidities during the year prior to the first health screening exam were defined using claims data and summarized using the Charlson comorbidity index [24]. Hypertension was defined as the presence of at least one I10–I13 or I15 code during the year prior to the screening exam, or a systolic blood pressure ≥140 mmHg or a diastolic blood pressure ≥90 mmHg at the baseline screening exam. Diabetes was defined as the presence of at least one E11–E14 code or a fasting glucose level of ≥126 mg/dL at the baseline screening exam. Dyslipidemia was defined as the presence of an E78 code or a total cholesterol level of > 240 mg/dL at the baseline screening exam.

Residential area at the time of the first screening exam was classified as metropolitan or rural. Metropolitan areas were defined as Seoul, 6 metropolitan cities, and 15 cities with population > 500,000 that have been officially designated as municipal cities (http://www.mois.go.kr). Data on income at the time of the first screening exam was obtained from the insurance eligibility database. Income level was categorized by percentile groups (≤30th, >30th–≤70th, and >70th percentile).

### Mortality follow-up

The NHIS-NSC 2.0 included mortality data from the Ministry of the Interior and Safety [19]. The study outcomes were liver-related mortality and all-cause mortality. Liver-related mortality was defined as liver cancer (code C22) or liver disease (codes B15–B19 or K70–K75) related mortality.

### Statistical analyses

Participants were followed-up from the time of their initial (baseline) health screening exam until death, 85 years of age, or December 31, 2015, whichever first. As age is a strong determinant of mortality, we used age as the time scale in the analyses [25]. Poisson regression was used to estimate marginally adjusted mortality rates by alcohol intake and ALT category. For risk analysis, we used Cox’s proportional hazard regression models to estimate hazard ratios (HRs) with 95% confidence intervals (CIs) for liver-related and all-cause mortality. Cox models were adjusted for sex, body mass index, Charlson comorbidity index, diabetes, hypertension, hyperlipidemia, smoking status (never, ever or unknown), residential area (metropolitan, rural and unknown), and income percentile (≤30th, >30th–≤70th, >70th, and unknown) [26]. We examined the assumption of proportional hazards using plots of the log(–log) survival function and Schoenfeld residuals. To examine the possibility of reverse causation, we conducted 2 sensitivity analyses: (1) excluding participants who died in the first 2 years of follow-up (*N*=2037), and (2) excluding participants with any existing chronic illnesses at baseline (1 Charlson comorbidity index ≥1, N = 116,830). A *p* value < 0.05 was considered statistically significant. All analyses were performed using SAS Enterprise Guide 7.1 (SAS Institute Inc., Cary, NC, USA) and Stata version 16 (StataCorp LP, College Station, TX, USA).

## Results

The mean (standard deviation) age of study participants (*N* = 367,612) was 52.4 (10.9) years and 46.9% of participants were men. The proportion of participants with elevated ALT levels was 23.4% (*N* = 85,877). Compared to participants with normal ALT levels, those with elevated levels were more likely to be male, obese, and have more co-morbidities. Among participants with normal ALT levels, the proportions of participants with no, light, and moderate alcohol intake were 58.8, 24.9, and 16.4%, respectively, while among participants with elevated ALT levels, the corresponding proportions were 57.4, 22.6, and 20.0%, respectively (Table 1).

**Table 1:** Baseline characteristics of study participants by alanine aminotransferase (ALT) level status (*N* = 367,612)

	Normal (281,735) ***N*** (%)	Elevated (85,877) ***N*** (%)	***p*** value
**Age, years**	52.5 (11.1)	52.2 (10.1)	< 0.01
**Sex,** male	128,266 (45.5)	44,021 (51.3)	< 0.01
**BMI, kg/m**^**2**^	23.5 (6.6)	25.4 (3.3)	< 0.01
**Waist circumference, cm**	79.6 (8.6)	84.6 (9.0)	< 0.01
**Creatinine**	1.0 (1.1)	1.0 (1.0)	0.83
**GGT, U/L**	20 (14-32)	41 (25-75)	< 0.01
**AST, U/L**	27.8 (8.5)	37.1 (23.6)	< 0.01
**Charlson comorbidity index**	0 (0–1)	0 (0–1)	< 0.01
**Diabetes**	30,807 (10.9)	16,209 (18.9)	< 0.01
**Hypertension**	84,112 (29.9)	35,053 (40.8)	< 0.01
**Hyperlipidemia**	111,175 (39.5)	52,561 (61.2)	< 0.01
**Alcohol amount**			< 0.01
None	165,552 (58.8)	49,312 (57.4)	
Light	70,075 (24.9)	19,404 (22.6)	
Moderate	46,108 (16.4)	17,161 (20.0)	
**Smoking status**			< 0.01
Never	183,564 (65.2)	51,571 (60.1)	
Ever	97,720 (34.7)	34,221 (39.8)	
Unknown	451 (0.2)	85 (0.1)	
**Residential area**			< 0.01
Metropolitan	181,399 (64.4)	54,553 (63.5)	
Rural	97,373 (34.6)	30,437 (35.4)	
Unknown	2963 (1.1)	887 (1.0)	
**Income percentile**			< 0.01
≤30th	66,944 (23.8)	19,454 (22.7)	
>30th–≤70th	94,719 (33.6)	30,008 (34.9)	
>70th	117,626 (41.8)	35,639 (41.5)	
Unknown	2446 (0.9)	776 (0.9)	

Abbreviation: *AST* aspartate aminotransferase, *BMI* body mass index, *GGT* gamma-glutamyl transferase

Normal: < 34 U/L for men and < 25 U/L for women; elevated: ≥34 U/L for men and ≥25 U/L for women

Values are presented as n (%) for categorical variables and mean (standard deviation) for continuous variables

**Fig. 2:**
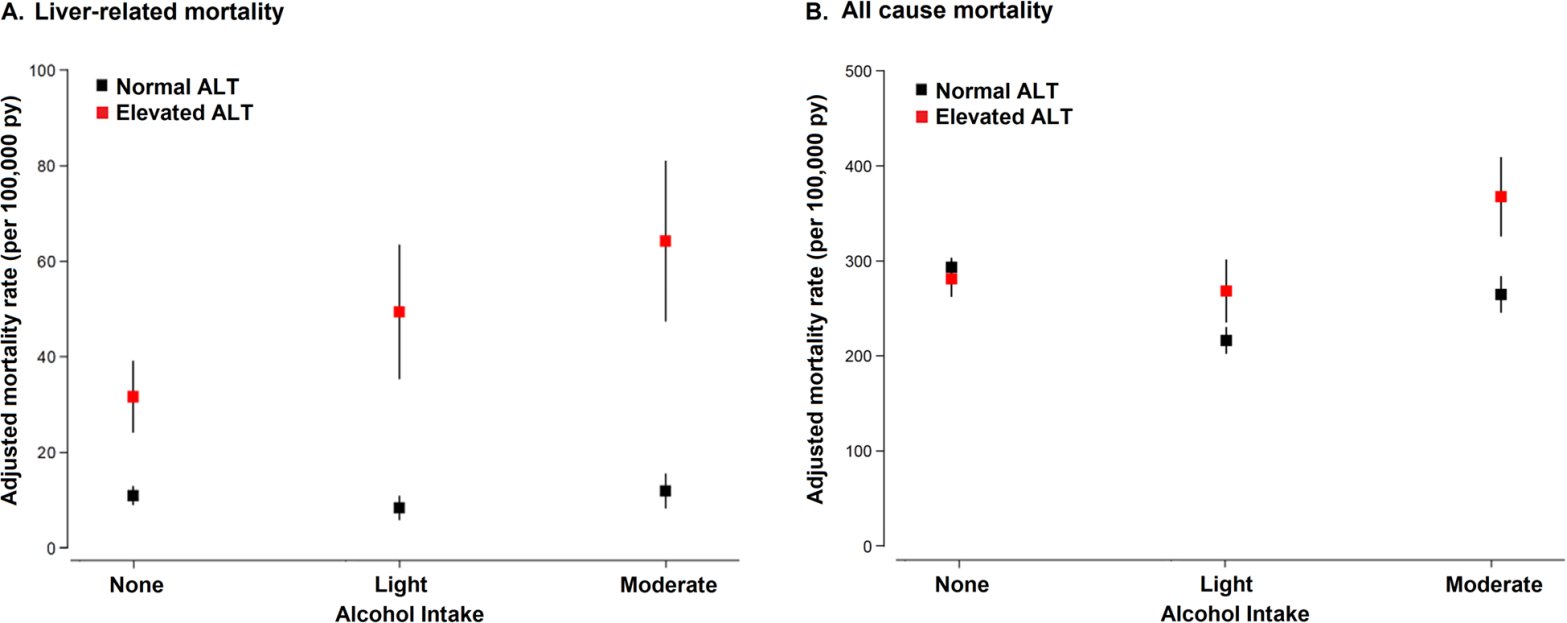
Multivariable-adjusted rates of liver-related mortality (**A**) and all-cause mortality (**B**) by alcohol intake and alanine aminotransferase (ALT) levels. Marginally-adjusted mortality rates were calculated from a Poisson regression model adjusted for age, sex, body mass index, Charlson comorbidity index, diabetes, hypertension, hyperlipidemia, smoking status (never, ever or unknown), residential area (metropolitan, rural and unknown), and income percentile (≤30th, >30th–≤70th, >70th, and unknown)

During 1,703,650 person-years of follow-up, we observed 7,020 (1.9%) deaths, including 401 liver-related deaths (0.1%). The fully-adjusted liver-related mortality rates in participants with normal and elevated ALT levels were 7 and 28 per 100,000 person-years, respectively (HR 4.07, 95% CI 3.32–5.01). Alcohol intake showed a positive association with liver-related mortality in participants with elevated ALT levels, but not in those with normal ALT levels (*p* value for alcohol intake by ALT interaction < 0.01; Table 2). Among those with elevated ALT levels, liver-related mortality increased with increasing category of alcohol intake (Table 2, Fig. 2).

**Table 2:** Multivariable-adjusted hazard ratios for liver-related and all-cause mortality associated with alcohol intake by alanine aminotransferase (ALT) level status

Alcohol intake	Alanine aminotransferase^**a**^		***p*** for interaction
	Normal HR (95% CI)	Elevated HR (95% CI)	
**Liver-related mortality**			< 0.01
None	*Reference*	*Reference*	
Light	0.73 (0.51, 1.05)	1.57 (1.08, 2.28)	
Moderate	1.06 (0.73, 1.52)	2.09 (1.46, 2.99)	
**All-cause mortality**			
None	*Reference*	*Reference*	< 0.01
Light	0.72 (0.66, 0.77)	0.93 (0.81, 1.08)	
Moderate	0.89 (0.82, 0.97)	1.31 (1.14, 1.50)	

HRs and 95% CIs were obtained from proportional hazards models with age as time scale and adjusted for sex, body mass index, Charlson comorbidity index, diabetes, hypertension, hyperlipidemia, smoking status (never, ever or unknown), residential area (metropolitan, rural, and unknown), and income percentile (≤30th, >30th–≤70th, >70th, and unknown)

Abbreviation: *HR*, hazard ratio; *CI*, confidence interval

^a^Normal: < 34 U/L for men and < 25 U/L for women; elevated: ≥34 U/L for men and ≥25 U/L for women

The fully-adjusted all-cause mortality rates in participants with normal and elevated ALT levels were 188 and 202 per 100,000 person-years, respectively (HR 1.08. 95% CI 1.02–1.15). In participants with normal ALT levels, the fully-adjusted HRs (95% CI) for all-cause mortality comparing light and moderate drinkers to non-drinkers were 0.72 (95% CI 0.66–0.77) and 0.89 (95% CI 0.82–0.97), respectively (Table 2 and Fig. 2). In participants with elevated ALT levels, the corresponding HRs were 0.93 (95% CI 0.81–1.08), and 1.31 (95% CI 1.14–1.50), respectively (*p* value for alcohol intake by ALT interaction < 0.01).

When we evaluated the HRs comparing elevated to normal ALT levels, the fully-adjusted HR for liver-related mortality comparing participants with elevated to those with normal ALT levels were 2.84 (95% CI 2.12–3.80), 6.10 (95% CI 1.03–9.24), and 5.63 (95% CI 3.79–8.34) for participants with none, light, and moderate of alcohol intake, respectively (Table 3). For all-cause mortality, the fully-adjusted HRs comparing participants with elevated to those with normal ALT levels were 0.97 (95% CI 0.90–1.04), 1.26 (95% CI 1.10–1.45), and 1.41 (95% CI 1.24–1.61) for participants with none, light, and moderate amount of alcohol intake, respectively (Table 3). In sensitivity analyses, the results were virtually the same (Additional file 1: Tables S1 and S2).

**Table 3:** Multivariable-adjusted hazard ratios for liver-related and all-cause mortality associated with elevated alanine aminotransferase (ALT) levels by alcohol intake status

Alcohol intake	Alanine aminotransferase^**a**^		***p*** for interaction
	Normal HR (95% CI)	Elevated HR (95% CI)	
**Liver-related mortality**			< 0.01
None	*Reference*	2.84 (2.12, 3.80)	
Light	*Reference*	6.10 (4.03, 9.24)	
Moderate	*Reference*	5.63 (3.79, 8.34)	
**All-cause mortality**			< 0.01
None	*Reference*	0.97 (0.90, 1.04)	
Light	*Reference*	1.26 (1.10, 1.45)	
Moderate	*Reference*	1.41 (1.24, 1.61)	

HRs and 95% CIs were obtained from proportional hazards models with age as time scale and adjusted for sex, body mass index, Charlson comorbidity index, diabetes, hypertension, hyperlipidemia, smoking status (never, ever or unknown), residential area (metropolitan, rural and unknown) and income percentile (≤30th, >30th–≤70th, >70th, and unknown)

Abbreviation: *HR*, hazard ratio; *CI*, confidence interval

^a^Normal: < 34 U/L for men and <25 U/L for women; elevated: ≥34 U/L for men and ≥25 U/L for women

## Discussion

In this large cohort study, we found that in individuals with elevated ALT levels, even small amounts of alcohol intake were associated with increased liver-related mortality and that a moderate amount of alcohol intake was associated with increased risk of all-cause mortality. This association was significantly different in subjects with normal ALT levels, in whom alcohol intake was not associated with increased liver-related or all-cause mortality. Our data provides support for recommending complete abstinence from alcohol in subjects with elevated ALT levels, as even a small amount of alcohol intake may be harmful to them.

Alcohol is a well-established liver toxicant and carcinogen [3, 27]. Even light and moderate alcohol intake can increase the risk of liver disease and of progression to advanced liver disease in individuals with liver disease [28–30]. Current evidence and practice guidelines recommend complete abstinence for patients with alcohol-associated liver disease and chronic viral hepatitis [10–13]. However, the health effects of light to moderate alcohol intake in patients with other liver diseases are not well established. In our data, we demonstrated the synergistic effect of alcohol intake and hepatic injury as measured by elevated serum ALT levels in individuals without alcohol-associated liver disease or chronic viral hepatitis. This suggests that individuals with elevated ALT levels should be advised total abstinence from alcohol, even if they are free of alcohol-associated liver disease or chronic viral hepatitis. In this study, light to moderate alcohol intake was associated with lower risk of all-cause mortality in subjects with normal ALT levels, while moderate alcohol intake was associated with increased risk of all-cause mortality in subjects with elevated ALT levels. The effect of light to moderate alcohol intake on health may thus differ depending on ALT levels. This may also explain some of the heterogeneity in the effects of small amounts of alcohol intake on health endpoints in previous studies [4–8].

There are some limitations to this study. The exact causes of elevated ALT levels in our study participants were unknown. The most common causes of elevated ALT levels in subjects undergoing health screening exams are alcohol intake, viral hepatitis, and NAFLD [18]. Since we excluded participants with heavy alcohol intake, chronic viral hepatitis, and rare forms of chronic liver disease (autoimmune, metabolic, or toxic liver disease), we expect NAFLD to be the cause of elevated ALT levels in most of our study participants. Furthermore, compared to participants without elevated ALT levels in our study, those with elevated ALT levels had a higher average BMI and a higher prevalence of diabetes, hypertension, and dyslipidemia, which are all key features of NAFLD. Unfortunately, NAFLD can only be identified when there is evidence of hepatic steatosis by histological or radiological evaluation in the absence of heavy alcohol intake and secondary causes for steatosis [31], and we lacked this information. Hence, participants with elevated ALT levels in this study do not exclusively represent subjects with NAFLD. In this study, we lacked follow-up ALT measurements and we could not determine whether the elevation in ALT levels was transient or persistent. Further studies with multiple ALT measurements are needed to ascertain whether light to moderate alcohol intake is harmful only for those with persistently elevated ALT levels or even for those with transient or intermittent elevated ALT levels. Information on alcohol intake was collected by self-administered questionnaires and was subject to measurement error and abstainer bias [32]. Since the questionnaire of the NHIS-NSC only asked current alcohol intake, we could not distinguish between never drinkers and former drinkers. Since some former drinkers may have stopped drinking because of health issues, including never drinkers and former drinkers in the reference category would tend to decrease the observed association of alcohol intake with mortality. In addition, we did not have information on the type or patterns of alcohol use [33], or on the change of alcohol use over time [34]. Our cohort was composed of Korean men and women participating in health screening exams. The effects of alcohol may differ by race/ethnicity [35] and genetic predisposition [36]. Hence, generalizability to other ethnic groups warrants further evaluation. Also, we cannot exclude the possibility of unmeasured or unknown confounding factors that were not controlled in the study. In this study, we used elevated ALT levels as the only indicator of liver injury. Further studies combining other biomarkers with ALT levels are needed to better identify and to quantify liver injury or liver disease. Strengths of this data include the use of representative nationwide data, which minimized the possibility of selection bias, the large sample size, and the availability of liver-specific and all-cause mortality information.

## Conclusions

In conclusion, a small amount of alcohol intake was associated with increased liver-related and all-cause mortality among individuals with elevated ALT levels. Subjects with elevated ALT levels should be advised complete abstinence from alcohol, as even a small amount of alcohol intake may be harmful for them.

## Supplementary Information

**Additional file 1: Table S1**. Multivariable-adjusted hazard ratios for liver-related and all-cause mortality associated with alcohol intake by alanine aminotransferase (ALT) level status in sensitivity analyses. **Table S2**. Multivariable-adjusted hazard ratios for liver-related and all-cause mortality associated with elevated alanine aminotransferase (ALT) levels by alcohol intake status in sensitivity analyses.

## Data Availability

We used the claim data provided by the Korean National Health Insurance Service (NHIS) database. Data can only be accessed by visiting the NHIS datacenter, after approval from data access committee of NHIS (https://nhiss.nhis.or.kr/bd/ab/bdaba001cv.do). This research was conducted using the application number NHIS-2019-2-034.

## Abbreviations

AASLD: The American Association for the Study of Liver Diseases
ALT: Alanine aminotransferase
BMI: Body mass index
CIs: Confidence intervals
HRs: Hazard ratios
NAFLD: Nonalcoholic fatty liver disease
NHIS-NSC: The National Health Insurance Service-National Sample Cohort

## References

[1] Kim BH, Park JW (2018). Epidemiology of liver cancer in South Korea. Clin Mol Hepatol.

[2] Park SH, Kim DJ (2020). Global and regional impacts of alcohol use on public health: Emphasis on alcohol policies. Clin Mol Hepatol.

[3] Iranpour A, Nakhaee N (2019). A Review of Alcohol-Related Harms: A Recent Update. Addict Health.

[4] Ronksley PE, Brien SE, Turner BJ, Mukamal KJ, Ghali WA (2011). Association of alcohol consumption with selected cardiovascular disease outcomes: a systematic review and meta-analysis. Bmj.

[5] Howard AA, Arnsten JH, Gourevitch MN (2004). Effect of alcohol consumption on diabetes mellitus: a systematic review. Ann Intern Med.

[6] Di Castelnuovo A, Costanzo S, Bagnardi V, Donati MB, Iacoviello L, de Gaetano G (2006). Alcohol dosing and total mortality in men and women: an updated meta-analysis of 34 prospective studies. Arch Intern Med.

[7] Holmes MV, Dale CE, Zuccolo L, Silverwood RJ, Guo Y, Ye Z, Prieto-Merino D, Dehghan A, Trompet S, Wong A, Cavadino A, Drogan D, Padmanabhan S, Li S, Yesupriya A, Leusink M, Sundstrom J, Hubacek JA, Pikhart H, Swerdlow DI, Panayiotou AG, Borinskaya SA, Finan C, Shah S, Kuchenbaecker KB, Shah T, Engmann J, Folkersen L, Eriksson P, Ricceri F, Melander O, Sacerdote C, Gamble DM, Rayaprolu S, Ross OA, McLachlan S, Vikhireva O, Sluijs I, Scott RA, Adamkova V, Flicker L, Bockxmeer FM, Power C, Marques-Vidal P, Meade T, Marmot MG, Ferro JM, Paulos-Pinheiro S, Humphries SE, Talmud PJ, Leach IM, Verweij N, Linneberg A, Skaaby T, Doevendans PA, Cramer MJ, Harst P, Klungel OH, Dowling NF, Dominiczak AF, Kumari M, Nicolaides AN, Weikert C, Boeing H, Ebrahim S, Gaunt TR, Price JF, Lannfelt L, Peasey A, Kubinova R, Pajak A, Malyutina S, Voevoda MI, Tamosiunas A, Maitland-van der Zee AH, Norman PE, Hankey GJ, Bergmann MM, Hofman A, Franco OH, Cooper J, Palmen J, Spiering W, Jong PA, Kuh D, Hardy R, Uitterlinden AG, Ikram MA, Ford I, Hypponen E, Almeida OP, Wareham NJ, Khaw KT, Hamsten A, Husemoen LLN, Tjonneland A, Tolstrup JS, Rimm E, Beulens JWJ, Verschuren WMM, Onland-Moret NC, Hofker MH, Wannamethee SG, Whincup PH, Morris R, Vicente AM, Watkins H, Farrall M, Jukema JW, Meschia J, Cupples LA, Sharp SJ, Fornage M, Kooperberg C, LaCroix AZ, Dai JY, Lanktree MB, Siscovick DS, Jorgenson E, Spring B, Coresh J, Li YR, Buxbaum SG, Schreiner PJ, Ellison RC, Tsai MY, Patel SR, Redline S, Johnson AD, Hoogeveen RC, Hakonarson H, Rotter JI, Boerwinkle E, PIW B, Kivimaki M, Asselbergs FW, Sattar N, Lawlor DA, Whittaker J, Davey Smith G, Mukamal K, Psaty BM, Wilson JG, Lange LA, Hamidovic A, Hingorani AD, Nordestgaard BG, Bobak M, Leon DA, Langenberg C, Palmer TM, Reiner AP, Keating BJ, Dudbridge F, Casas JP, on behalf of The InterAct Consortium (2014). Association between alcohol and cardiovascular disease: Mendelian randomisation analysis based on individual participant data. BMJ.

[8] Pimpin L, Cortez-Pinto H, Negro F, Corbould E, Lazarus JV, Webber L, Sheron N, EASL HEPAHEALTH Steering Committee (2018). Burden of liver disease in Europe: Epidemiology and analysis of risk factors to identify prevention policies. J Hepatol.

[9] Cederbaum AI (2012). Alcohol metabolism. Clin Liver Dis.

[10] Crabb DW, Im GY, Szabo G, Mellinger JL, Lucey MR (2020). Diagnosis and Treatment of Alcohol-Associated Liver Diseases: 2019 Practice Guidance From the American Association for the Study of Liver Diseases. Hepatology.

[11] Sinn DH, Kang D, Guallar E, Chang Y, Ryu S, Zhao D, Hong YS, Cho J, Gwak GY (2021). Alcohol Intake and Mortality in Patients With Chronic Viral Hepatitis: A Nationwide Cohort Study. Am J Gastroenterol.

[12] Korean Association for the Study of the Liver (2018). 2017 KASL clinical practice guidelines management of hepatitis C: Treatment of chronic hepatitis C. Clin Mol Hepatol.

[13] Korean Association for the Study of the Liver (2019). KASL clinical practice guidelines for management of chronic hepatitis B. Clin Mol Hepatol.

[14] Fuster D, Samet JH (2018). Alcohol Use in Patients with Chronic Liver Disease. N Engl J Med.

[15] Pratt DS, Kaplan MM (2000). Evaluation of abnormal liver-enzyme results in asymptomatic patients. N Engl J Med.

[16] Newsome PN, Cramb R, Davison SM, Dillon JF, Foulerton M, Godfrey EM, Hall R, Harrower U, Hudson M, Langford A, Mackie A, Mitchell-Thain R, Sennett K, Sheron NC, Verne J, Walmsley M, Yeoman A (2018). Guidelines on the management of abnormal liver blood tests. Gut.

[17] Green RM, Flamm S (2002). AGA technical review on the evaluation of liver chemistry tests. Gastroenterology.

[18] Park HN, Sinn DH, Gwak GY, Kim JE, Rhee SY, Eo SJ, Kim YJ, Choi MS, Lee JH, Koh KC, Paik SW, Yoo BC (2012). Upper normal threshold of serum alanine aminotransferase in identifying individuals at risk for chronic liver disease. Liver Int.

[19] Lee J, Lee JS, Park SH, Shin SA, Kim K (2017). Cohort Profile: The National Health Insurance Service-National Sample Cohort (NHIS-NSC). South Korea Int J Epidemiol.

[20] Health Insurance Review Assessment Service (2010). National Health Insurance Statistical Yearbook.

[21] Chun C-B, Kim S-Y, Lee J-Y, Lee S-Y. Republic of Korea: Health system review. Health Syst Trans. 2009;11(7):1–184.

[22] Shin DW, Cho B, Guallar E (2016). Korean National Health Insurance Database. JAMA Intern Med.

[23] Kim MK, Lee WY, Kang JH, Kang JH, Kim BT, Kim SM, Kim EM, Suh SH, Shin HJ, Lee KR, Lee KY, Lee SY, Lee SY, Lee SK, Lee CB, Chung S, Jeong IK, Hur KY, Kim SS, Woo JT, Committee of Clinical Practice Guidelines, Korean Society for the Study of Obesity (2014). 2014 clinical practice guidelines for overweight and obesity in Korea. Endocrinol Metab (Seoul).

[24] Charlson ME, Pompei P, Ales KL, MacKenzie CR (1987). A new method of classifying prognostic comorbidity in longitudinal studies: development and validation. J Chronic Dis.

[25] Thiebaut AC, Benichou J (2004). Choice of time-scale in Cox's model analysis of epidemiologic cohort data: a simulation study. Stat Med.

[26] Acs G (2011). Defining the Middle Class. Downward Mobility from the Middle Class: Waking Up from the American Dream.

[27] Hsu CC, Kowdley KV (2016). The Effects of Alcohol on Other Chronic Liver Diseases. Clin Liver Dis.

[28] Chang Y, Ryu S, Kim Y, Cho YK, Sung E, Kim HN, Ahn J, Jung HS, Yun KE, Kim S, Sung KC, Sohn CI, Shin H, Wild SH, Byrne CD (2020). Low Levels of Alcohol Consumption, Obesity, and Development of Fatty Liver With and Without Evidence of Advanced Fibrosis. Hepatology.

[29] Chang Y, Cho YK, Kim Y, Sung E, Ahn J, Jung HS, Yun KE, Shin H, Ryu S (2019). Nonheavy Drinking and Worsening of Noninvasive Fibrosis Markers in Nonalcoholic Fatty Liver Disease: A Cohort Study. Hepatology.

[30] Aberg F, Puukka P, Salomaa V, Mannisto S, Lundqvist A, Valsta L (2020). Risks of Light and Moderate Alcohol Use in Fatty Liver Disease: Follow-Up of Population Cohorts. Hepatology.

[31] Wong VW, Chan WK, Chitturi S, Chawla Y, Dan YY, Duseja A (2018). Asia-Pacific Working Party on Non-alcoholic Fatty Liver Disease guidelines 2017-Part 1: Definition, risk factors and assessment. J Gastroenterol Hepatol.

[32] Rehm J, Spuhler T (1993). Measurement error in alcohol consumption: the Swiss Health Survey. Eur J Clin Nutr.

[33] Mitchell T, Jeffrey GP, de Boer B, MacQuillan G, Garas G, Ching H, Hamdorf J, Adams LA (2018). Type and Pattern of Alcohol Consumption is Associated With Liver Fibrosis in Patients With Non-alcoholic Fatty Liver Disease. Am J Gastroenterol.

[34] Gronbaek M, Johansen D, Becker U, Hein HO, Schnohr P, Jensen G (2004). Changes in alcohol intake and mortality: a longitudinal population-based study. Epidemiology.

[35] Kerr WC, Greenfield TK, Bond J, Ye Y, Rehm J (2011). Racial and ethnic differences in all-cause mortality risk according to alcohol consumption patterns in the national alcohol surveys. Am J Epidemiol.

[36] Stickel F, Lutz P, Buch S, Nischalke HD, Silva I, Rausch V, Fischer J, Weiss KH, Gotthardt D, Rosendahl J, Marot A, Elamly M, Krawczyk M, Casper M, Lammert F, Buckley TWM, McQuillin A, Spengler U, Eyer F, Vogel A, Marhenke S, Felden J, Wege H, Sharma R, Atkinson S, Franke A, Nehring S, Moser V, Schafmayer C, Spahr L, Lackner C, Stauber RE, Canbay A, Link A, Valenti L, Grove JI, Aithal GP, Marquardt JU, Fateen W, Zopf S, Dufour JF, Trebicka J, Datz C, Deltenre P, Mueller S, Berg T, Hampe J, Morgan MY (2020). Genetic Variation in HSD17B13 Reduces the Risk of Developing Cirrhosis and Hepatocellular Carcinoma in Alcohol Misusers. Hepatology.

