# Supplementary material for: Modest alcohol intake and mortality in individuals with elevated alanine aminotransferase levels: a nationwide cohort study
Source: BMC Med. 2022 Jan 24;20:18. doi: 10.1186/s12916-021-02215-x (PMC8785562; doi:10.1186/s12916-021-02215-x)
Supplement: Supplementary file 1 — Additional file 1: Table S1. Multivariable-adjusted hazard ratios for liver-related and all-cause mortality associated with alcohol intake by alanine aminotransferase (ALT) level status in sensitivity analyses. Table S2. Multivariable-adjusted hazard ratios for liver-related and all-cause mortality associated with elevated alanine aminotransferase (ALT) levels by alcohol intake status in sensitivity analyses. [file 12916_2021_2215_MOESM1_ESM.docx]

**Table S1.** Multivariable-adjusted hazard ratios for liver-related and all-cause mortality associated with alcohol intake by alanine aminotransferase (ALT) level status in sensitivity analyses.

| **Alcohol intake** | **Alanine aminotransferase^a^** | | ***p* for interaction** |
| --- | --- | --- | --- |
|  | **Normal**  **HR (95% CI)** | **Elevated**  **HR (95% CI)** |  |
| **Excluding participants who died in the first 2 years (N = 365,575)** | | | |
| **Liver-related mortality** |  |  | 0.01 |
| None | *Reference* | *Reference* |  |
| Light | 0.86 (0.58, 1.28) | 1.74 (1.12, 1.80) |  |
| Moderate | 1.19 (0.79, 1.80) | 2.47 (1.61, 3.78) |  |
| **All-cause mortality** |  |  |  |
| None | *Reference* | *Reference* | < 0.01 |
| Light | 0.76 (0.70, 0.83) | 1.03 (0.87, 1.22) |  |
| Moderate | 0.95 (0.86, 1.05) | 1.51 (1.29, 1.77) |  |
| **Excluding participants who died in the first 2 years and had ≥ 1 of Charlson comorbidity index (N = 249,862)** | | | |
| **Liver-related mortality*** |  |  | < 0.01 |
| None | *Reference* | *Reference* |  |
| Light | 0.82 (0.49, 1.36) | 2.60 (1.43, 4.74) |  |
| Moderate | 0.92 (0.53, 1.60) | 2.84 (1.56, 5.16) |  |
| **All-cause mortality*** |  |  |  |
| None | *Reference* | *Reference* | < 0.01 |
| Light | 0.76 (0.67, 0.86) | 1.18 (0.93, 1.50) |  |
| Moderate | 0.94 (0.82, 1.07) | 1.69 (1.36, 2.10) |  |

*Abbreviation:* HR, hazard ratio; CI, confidence interval

HRs and 95% CIs were obtained from proportional hazards models with age as time scale and adjusted for sex, body mass index, Charlson comorbidity index, diabetes, hypertension, hyperlipidemia, smoking status (never, ever or unknown), residential area (metropolitan, rural and unknown) and income percentile (≤30^th^, >30^th^–≤70^th^, >70^th^, and unknown)

*The model was not adjusted for Charlson comorbidity index as participants who had at least 1 Charlson comorbidity were excluded in the model.

^a^Normal: <34 U/L for men and <25 U/L for women; elevated: ≥34 U/L for men and ≥25 U/L for women

**Table S2.** Multivariable-adjusted hazard ratios for liver-related and all-cause mortality associated with elevated alanine aminotransferase (ALT) levels by alcohol intake status in sensitivity analyses.

| **Alcohol intake** | **Alanine aminotransferase^a^** | | ***p* for interaction** |
| --- | --- | --- | --- |
|  | **Normal**  **HR (95% CI)** | **Elevated**  **HR (95% CI)** |  |
| **Excluding participants who died in the first 2 years (N = 365,575)** | | | |
| **Liver-related mortality** | | | < 0.01 |
| None | *Reference* | 2.43 (1.73, 3.43) |  |
| Light | *Reference* | 4.94 (3.09, 7.92) |  |
| Moderate | *Reference* | 5.03 (3.21, 7.88) |  |
| **All-cause mortality** | | | < 0.01 |
| None | *Reference* | 0.93 (0.85, 1.02) |  |
| Light | *Reference* | 1.26 (1.07, 1.48) |  |
| Moderate | *Reference* | 1.47 (1.26, 1.72) |  |
| **Excluding participants who died in the first 2 years and had ≥ 1 of Charlson comorbidity index (N = 249,862)** | | | |
| **Liver-related mortality*** | | | < 0.01 |
| None | *Reference* | 1.91 (1.14, 3.20) |  |
| Light | *Reference* | 6.08 (3.43, 10.76) |  |
| Moderate | *Reference* | 5.86 (3.27, 10.52) |  |
| **All-cause mortality*** | | | < 0.01 |
| None | *Reference* | 0.89 (0.77, 1.03) |  |
| Light | *Reference* | 1.39 (1.12, 1.74) |  |
| Moderate | *Reference* | 1.61 (1.32, 1.96) |  |

*Abbreviation:* HR, hazard ratio; CI, confidence interval

HRs and 95% CIs were obtained from proportional hazards models with age as time scale and adjusted for sex, body mass index, Charlson comorbidity index, diabetes, hypertension, hyperlipidemia, smoking status (never, ever or unknown), residential area (metropolitan, rural and unknown) and income percentile (≤30^th^, >30^th^–≤70^th^, >70^th^, and unknown)

*The model was not adjusted for Charlson comorbidity index as participants who had at least 1 Charlson comorbidity were excluded in the model.

^a^Normal: <34 U/L for men and <25 U/L for women; elevated: ≥34 U/L for men and ≥25 U/L for women
